# Wernicke Encephalopathy Following Severe Hyperemesis Gravidarum: A Missed Opportunity for Timely Thiamine Replacement

**DOI:** 10.7759/cureus.87478

**Published:** 2025-07-07

**Authors:** Risq Atiqah Munirah Mohamed Mustafa, Noor Azah Abd Aziz, Ahmad Fithri Azam Abdul Rahman

**Affiliations:** 1 Department of Family Medicine, Faculty of Medicine, Universiti Kebangsaan Malaysia Medical Centre, Kuala Lumpur, MYS; 2 Family Medicine, Klinik Kesihatan Ayer Keroh, Malacca City, MYS

**Keywords:** hyperemesis gravidarum (hg), pregnant, primary care, thiamine, wernicke encephalopathy (we)

## Abstract

Wernicke’s encephalopathy (WE) is a rare but potentially fatal complication of hyperemesis gravidarum (HG) caused by thiamine (vitamin B1) deficiency. WE is a medical emergency with complete recovery occurring in only a few cases associated with HG in pregnancy. Classical clinical features include the triad of ophthalmoplegia, altered mental status, and ataxia.

We report a case of a 42-year-old gravida 2 para 1 female patient at 16 weeks and 3-day period of gestation (POG) who presented to primary care during routine antenatal follow-up with a two-week history of sudden visual loss. She had experienced persistent nausea and vomiting for approximately eight weeks and reported a total weight loss of 15 kg since early pregnancy, including 5 kg in the past two weeks. At presentation, she appeared cachectic and extremely lethargic. She was tachycardic with a heart rate (HR) of 116 beats per minute, though her blood pressure remained normotensive. Eye examination revealed visual acuity of counting fingers with positive horizontal nystagmus, suggesting ophthalmoplegia. Fundoscopy was normal.

Urinalysis showed ketonuria and proteinuria, with laboratory findings showing severe acute kidney injury (AKI). She was promptly referred to a tertiary center where she was treated with intravenous thiamine 500mg three times daily along with electrolyte replacement and hydration. Her symptoms improved rapidly, and her visual acuity returned to 6/6 bilaterally within 48 hours. She was discharged on a tapering dose of oral thiamine, which was to be continued until delivery, and remained well throughout the rest of her pregnancy. At 28 weeks' gestation, she underwent an emergency caesarean section due to fetal distress and delivered a premature baby girl weighing 1.3 kg.

This case highlights the need for early recognition of WE in pregnant women with severe HG. Although rare, it is a reversible complication. Timely thiamine supplementation is essential for both treatment and prevention, and prophylaxis should be considered in high-risk patients.

## Introduction

WE is an emergency condition but potentially reversible neurological syndrome resulting from thiamine also known as vitamin B1 deficiency [1]. Thiamine plays a crucial role in several biochemical pathways involved in energy metabolism [2]. Its requirement increases in conditions that elevate metabolic demand and glucose intake, such as in childhood, critical illnesses, malignancy, pregnancy, lactation, and with high-calorie intake. The recommended daily thiamine intake is 0.4mg per 1000kcal per day, increasing to 1.5 mg during pregnancy [3]. The body’s thiamine stores are typically sufficient for approximately 18 days, making individuals with prolonged nutritional imbalance for more than two to three weeks susceptible to deficiency [4]. The underlying neuronal damage in thiamine deficiency is attributed to mitochondrial dysfunction, oxidative stress, and cell apoptosis [2].

WE is often underdiagnosed, with 75-80% of cases initially missed [2]. Though classically linked to chronic alcohol use, WE can occur in various non-alcoholic settings, including severe hyperemesis gravidarum (HG), anorexia nervosa, and post-bariatric surgery [2,5]. It may develop in about 0.1-0.5% of severe HG cases; hence, prompt diagnosis requires a high degree of suspicion by the clinician [6]. Clinically, about 66% of patients present with the classic triad: confusion with temporospatial disorientation (80%), ataxia (76%), and ophthalmoplegia (93%) [1]. Persistent vomiting in HG causes dehydration, electrolyte imbalance, and weight loss, increasing the risk of thiamine depletion and subsequent WE. Unfortunately, there is no specific laboratory blood test to confirm WE. Diagnosis is primarily clinical, but imaging may support the diagnosis and help to exclude other conditions. Brain CT scans have low sensitivity but help exclude alternative pathologies. MRI offers higher sensitivity and specificity (up to 93%), often showing symmetrical hyper-intense lesions on T2-weighted and FLAIR sequences, changes mainly in the thalamus, mamillary bodies, tectal plate, and periaqueductal area [7].

Management of WE requires prompt parenteral thiamine administration before any glucose infusion, as glucose can worsen neuronal injury [2]. Early recognition and treatment are crucial to prevent progression. Untreated or delayed WE can lead to irreversible complications such as Korsakoff syndrome, characterised by severe memory deficits, confabulation, and learning disabilities [7]. This case report highlights the importance of early thiamine supplementation in prolonged HG, emphasising the role of multidisciplinary care involving both hospital and primary care providers.

## Case presentation

A 42-year-old Malay lady, gravida 2 para 1, presented for routine antenatal follow-up at 16 weeks of gestation. Her chief complaint was having a sudden-onset bilateral blurring of vision for the past two weeks. She reported that her visual acuity had been deteriorating to the extent that she could only perceive shadows and required assistance with ambulation and daily activities. Despite having visual symptoms and extreme fatigue, she did not seek medical attention earlier, opting instead to wait until her scheduled clinic visit, as she thought it was not a significant concern. 

Four weeks before this current visit, she had been admitted to the hospital for nausea and vomiting in pregnancy complicated by a urinary tract infection (UTI). However, she discharged herself against medical advice after a brief admission. Following that, she experienced persistent non-projectile vomiting around two to three episodes per day, consisting mainly of food particles and gastric contents without hematemesis. Her oral intake was severely compromised; she was only able to tolerate sweetened beverages and fruit juices and would vomit after consuming even small amounts of solid food. She had a significant weight loss of 15kg, from a pre-pregnancy weight of 62 kg to 47 kg, with a recent 5 kg loss in the two weeks prior to this current clinic visit.

She described profound generalized weakness and lethargy, remaining mostly bedbound during this period. Her husband and mother-in-law assisted her with basic needs, including toileting and feeding. Despite her deteriorating condition, she denied symptoms of hyperthyroidism such as palpitations, tremor, heat intolerance, or diarrhea. She also reported no headache to suggest increase intracranial pressure (ICP), fever, abdominal pain, urinary incontinence or per vaginal discharge or bleeding. 

Upon presentation in the primary care clinic, she appeared cachectic with sunken eyes. Her tongue was dry and coated, but still pink, and skin turgor was preserved. Capillary refill time was less than two seconds. Her vital signs were stable she was afebrile, blood pressure was normotensive with 118/89 mmHg, heart rate of 116 beats per minute with regular rhythm, and normal pulse volume. Her Glasgow Coma Scale (GCS) was 15/15. Abdominal examination revealed a soft, non-tender abdomen with her uterus palpable at 16 weeks' size. Bilateral renal punch was negative. Neurological examination showed normal tone, power, and reflexes in both bilateral upper and lower limbs. However, horizontal nystagmus was observed, though there was no diplopia, and the relative afferent pupillary defect (RAPD) was negative. Given her visual complaints, she was urgently referred to the emergency department for further evaluation. The chronology of events is as described in Figure 3.

**Figure 1:**
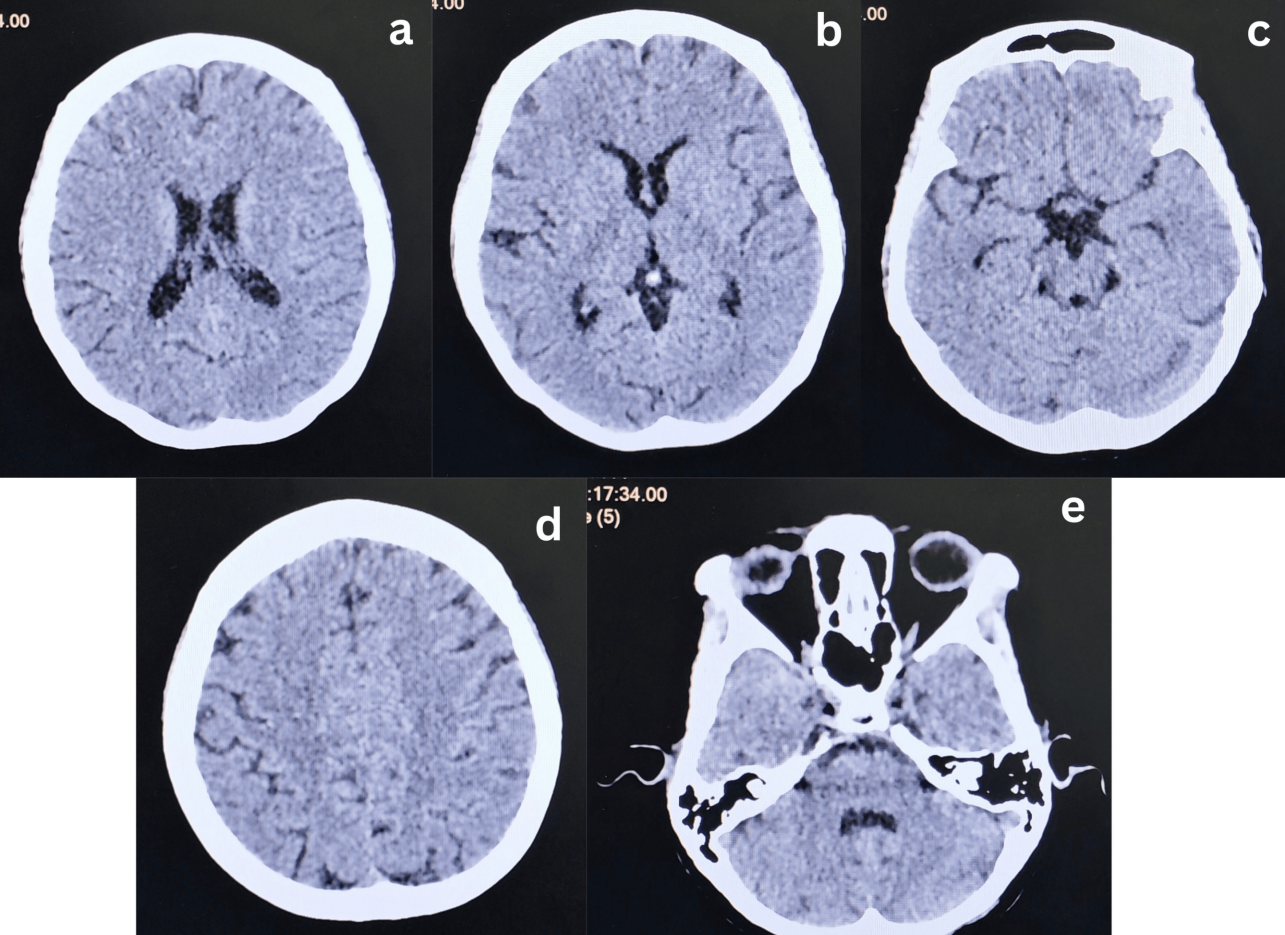
Axial non-contrast CT brain showing no acute hemorrhage, infarction, or mass effect. Normal appearance of the ventricles and cerebral structures. Findings are non-specific. a: Level of corona radiata, b: level of basal ganglia and 3rd ventricle, c: level of basal cistern, d: level of centrum semi-ovale, e: level of 4th ventricle and cerebellum

**Figure 2:**
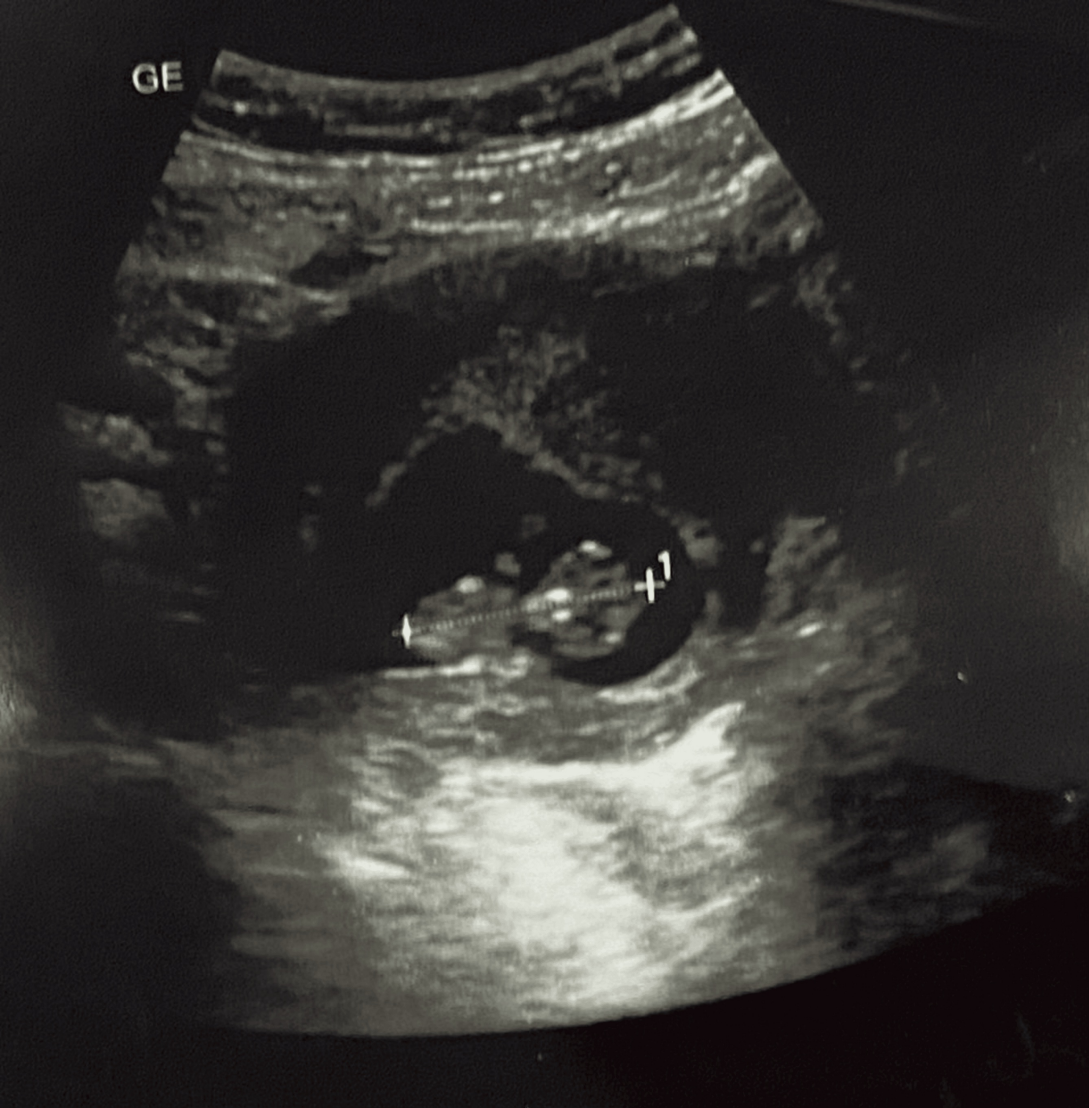
Figure shows early dating obstetric ultrasound scan of the patient at 9 weeks and 6 days period of gestation (POG) which reveals a singleton viable intrauterine pregnancy. Crown-rump-length measurements were 2.99 cm and given a revised estimated due date (REDD). No fetal anomalies were detected.

In the emergency department, an urgent CT Brain was performed to rule out a space-occupying lesion, and the result was unremarkable (Figure 1). Ophthalmological assessment by ophthalmologists revealed significant visual impairment bilaterally (Table 1). Laboratory investigations demonstrated features of dehydration and electrolyte imbalance with ketonuria (4+), glycosuria (1+), proteinuria (3+), and positive nitrites and leukocytes on urinalysis. However, urine culture revealed no significant growth. Her renal function tests showed acute kidney injury (Table 2). Liver and thyroid function tests were within normal limits (Free T4, 18.8 pmol/L, thyroid-stimulating hormone (TSH) 1.63 mIU/L). Serial transabdominal scan showed a singleton fetus with visualized fetal heart and parameters corresponding to date (Figure 2 & Table 3). Gross anomaly scan done by an obstetrician at 20 weeks' gestation also reveals a grossly normal fetus.

**Table 1:** Eye assessment at emergency department by ophthalmology team

Right	Examination	Left
Hand movement	Visual acuity	Counting fingers
Pupil reactive 3/3, cornea clear, A/C deep, white conjunctiva and clear lens	Anterior segment of the eye	Pupil reactive 3/3, cornea clear, A/C deep, white conjunctiva and clear lens
Pink, not swollen	Optic disc	Pink, not swollen
0.3	CDR	0.3
Intact	Macula	Intact
Normal	Fundus examination	Normal
No retinitis/vasculitis/ hemorrhage	Others	No retinitis/vasculitis/ hemorrhage

**Table 2:** Renal function test

Date:	Baseline	Admission	In ward	Discharge	Normal value
Urea (mmol/L)	2.4	9.8	8.9	3.1	1.7-8.3
Sodium (mmol/L)	136	132	137	137	135-145
Potassium (mmol/L)	3.5	4.9	3.7	3.4	3.5-5.0
Creatinine (umol/L)	48	114	82	59	44-80
eGFR	117.4	51.6	76.9	109.7	ml/min/1.73m2

**Table 3:** Serial trans-abdominal scan for fetal growth monitoring w, week; d, day; BPD: biparietal diameter; HC: head circumference; AC: abdominal circumference; FL: fetal length; EFW: estimated fetal weight; FH: fetal heart; DVP: Deep Vertical Pool; AFI: Amniotic Fluid Index *Exact measurements were not provided, as the sonographer reported approximate values. Parameters are based on the overall assessment of the fetus.

Gestational age	16w+3d	20w+3d	24w+1d	28w
Parameters	15w-17w	19w-21w	22w-24w	25-26w
Presentation	Singleton, transverse	Singleton, cephalic	Singleton, cephalic	Singleton, cephalic
BPD	2.97cm (15w+3d)	(19w-21w)*	5.59 cm (23w+0d)	(25-26 w)*
HC	12.41 cm (16w+4d)	(19-21 w)*	20.51 cm (22w+4d)	(25-26 w)*
AC	10.58 cm (16w+4d)	(19-21 w)*	17.17 cm (22w+1d)	(25-26 w)*
FL	1.75 cm (15w+1d)	(19-21 w)*	3.89 cm (22 w + 1d)	(25-26 w)*
EFW	137.94 g	292.34 g	496.77 g	888.00 g
Placenta l*ocation	Anterior, not low-lying	Anterior, not low-lying	Anterior, not low-lying	Placenta anterior upper segment
FH	Present	Present	Present	Present
DVP/ AFI	Adequate	Adequate	DVP: 3.54cm	AFI: 13.8cm
Remarks	Corresponds to date	Gross anomalies scan, normal	Corresponds to date	Doppler: RI: 0.56; PI: 0.77

A diagnosis of Wernicke encephalopathy (WE) was made clinically based on the symptoms and signs of ophthalmoplegia (nystagmus) and ataxia (gait disturbance requiring assistance). Though not all symptoms were overt, and she was still oriented, notably, her WE diagnosis was delayed, as initially she was admitted to rule out a space-occupying lesion (SOL). Prior to the diagnosis of WE, she was given hydration with glucose supplementation and normal saline without thiamine administration; however, her glucose solution was stopped upon diagnosis of WE.

Following the diagnosis, she was promptly initiated on intravenous (IV) thiamine 500 mg three times daily for five days. Within 48 hours of thiamine therapy, her visual acuity improved significantly and normalized to 6/6 bilaterally. Her thiamine supplementation was then subsequently titrated down gradually to oral dosing: 100mg daily for one week, followed by 50mg, 40 mg, 30 mg, 20 mg daily for a week, with maintenance therapy of 10 mg oral thiamine daily for the remainder of the pregnancy. She was also concurrently treated with prophylactic subcutaneous enoxaparin (Clexane) 40mg once daily for venous thromboembolism prevention, in line with HG management. A diagnosis of UTI was also confirmed and managed with the given oral cefuroxime 250 mg twice daily for one week.

The patient showed significant clinical improvement. Her nausea and vomiting subsided, her oral intake improved, and ketonuria resolved. She was discharged after 12 days of admission with complete resolution of visual symptoms and improved general condition. At 28 weeks of gestation, she delivered a baby girl prematurely via emergency caesarean section due to fetal distress, weighing 1.3 kg. She remained well throughout the post-partum period, and the baby was thriving well after admission to the neonatal intensive care unit for one month and subsequently discharged. 

**Figure 3:**
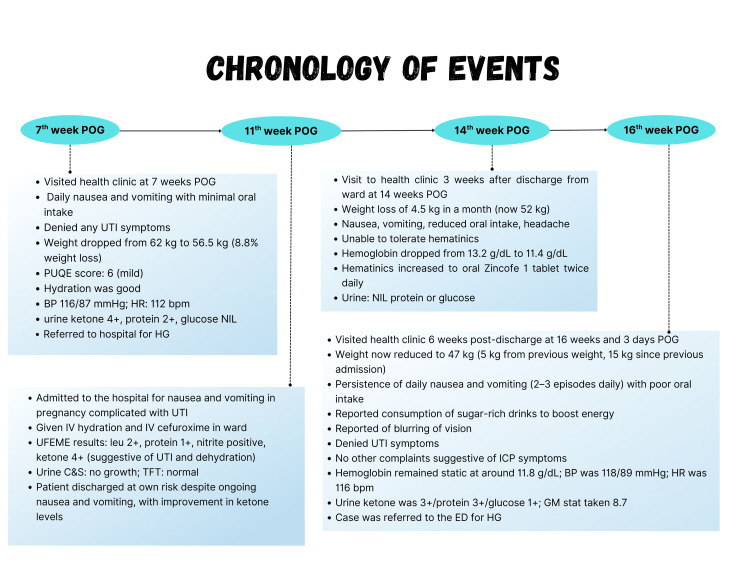
Chronology of events

## Discussion

This case report highlights the critical need for clinical vigilance in detecting Wernicke's encephalopathy (WE) in pregnant women with HG, especially when the presentation is atypical. Our patient, an advanced maternal age antenatal lady, developed visual disturbances as the earliest and sole neurological symptom, contributing to a delay in recognizing this life-threatening yet preventable condition. While the classic triad of WE - ophthalmoplegia, ataxia, and altered mental status - is well documented, it is often incomplete, and up to 19% patients may initially exhibit only one of these symptoms. A 2021 systematic review by Oudman et al. found that only 16% of pregnant patients with WE presented with all three classic signs, with ocular symptoms being most common (82%), and mental status changes presented in only 47% of cases [7]. This underlines the diagnostic challenge posed by the variable and subtle onset of WE, particularly when symptoms are misattributed to benign pregnancy-related causes due to dehydration or fatigue [8]. ** **

Diagnosis of WE remains largely clinical, as thiamine levels are not routinely available and may not accurately reflect tissue stores [6]. Imaging, although supportive, has low sensitivity in the early stages, with MRI being the best modality to detect abnormalities due to thiamine deficiency, further complicating diagnosis [7]. This was evident in our patient, whose initial imaging with CT brain was inconclusive. Thus, clinical judgement and a high index of suspicion remain paramount, especially in patients with ongoing vomiting, weight loss, and cognitive or ocular symptoms. The implications of delayed recognition are significant; fetal loss may approach 50%, maternal mortality can reach 5% and up to 65.4% of patients may progress to Korsakoff's syndrome, a long-term cognitive impairment characterized by severe amnesia, executive problems, and confabulation. It usually developed in patients who had more acute symptoms of the classical triad (median of 2.8 symptoms) [7]. Although our patient and baby were thriving well, the risk of fetal loss is there, as our patient delivered prematurely at 28 weeks. 

This case also brings attention to the critical role of primary care in the early identification and prevention of nutritional deficiencies during pregnancy. Thiamine deficiency in HG can develop between 10-15 weeks of gestation, with a median presentation of seven weeks of vomiting (ranging from 1-30 weeks of vomiting) [7]. Systematic review on cases of WE in pregnancy revealed a mean loss of weight due to HG was 12.1kg (ranging from 2-20 kg of weight lost among patients) [7]. HG patient frequently develops maladaptive dietary patterns, as seen in our patient, such as relying on sweet beverages or limiting intake due to nausea, which further reduces thiamine intake. The risk is also exacerbated by the administration of dextrose-containing fluids prior to thiamine repletion, which can worsen lactic acidosis and precipitate neurological damage. This iatrogenic harm, observed in our patient, is well-established in the literature and further emphasizes the need for guideline adherence [1,3,7]. While Malaysian guidelines recommend early thiamine supplementation in at-risk patients with prolonged vomiting, these guidelines are inconsistently applied in both primary and secondary care [9]. Patients should also be encouraged to consume small, frequent meals high in protein and low in fat, and avoid dietary triggers that exacerbate nausea. 

Thiamine supplementation is a gold standard treatment for WE [4]. Current recommendations suggest administering 100 mg of intravenous or intramuscular thiamine daily in patients with persistent vomiting, weight loss, or suspected nutritional deficiencies which at risk of developing WE for three to five days, followed by oral thiamine 100 mg three times daily for one to two weeks and 100 mg daily thereafter [7]. This patient missed this opportunity for early thiamine supplementation during her hospital admission prior to WE onset, which could have prevented the patient from developing WE. Picking up this in primary care for early thiamine supplementation, when she was still vomiting and losing weight continuously, may have improved the outcome. 

On the other hand, in cases where WE are already suspected, a higher dosage of intravenous thiamine 500 mg three times daily is recommended for at least five to seven days or until symptoms improve as projected in this case followed by oral thiamine 100 mg three times daily for one to two weeks and 100 mg daily subsequently [1,3]. Lack of consensus guidelines can lead to suboptimal treatment with thiamine supplementation, causing them to be susceptible to developing persistent cognitive problems due to Korsakoff’s syndrome [7]. The treatment with thiamine is lifesaving and has the potential to reverse this acute neuropsychiatric syndrome.

Improvements can be made at multiple levels. In primary care, standardized antenatal records should include screening questions for nutritional risk, vomiting severity, and dietary patterns. Home care is also a critical component in managing high-risk antenatal patients with HG to prevent severe complications like WE. Oral thiamine supplementation should be initiated in any HG patient unable to maintain adequate intake for more than a week, especially if home-managed. Healthcare providers must be educated on the red flags of WE, such as blurring of vision, ataxia, and confusion, and the critical sequences of thiamine administration before any glucose-containing fluids. In tertiary settings, protocols should mandate empirical IV Thiamine for all HG patients with neurological symptoms, regardless of lab or imaging findings. As projected in Oudman et al.'s review, early thiamine supplementation resulted in full recovery in over 80% of cases, whereas delayed treatment led to irreversible cognitive deficits or fetal demise [7]. 

On a system level, proper guidelines in national maternal health policies [10] and developing antenatal education materials for at-risk patients can significantly improve outcomes. A national registry of WE in pregnancy could also help quantify its true burden and highlight missed opportunities for early intervention. This case report highlights the missed prevention opportunities at the primary care level and serves as a cautionary tale from obstetric and primary care practice. It reflects on how subtle neurological complaints in antenatal patients can mask an evolving encephalopathy and why structured, anticipatory care, including early thiamine supplementation, timely referral, and improved provider training must be prioritized. Telemedicine can also play a significant role by enabling remote assessments, providing dietary guidance, and allowing timely adjustments to treatment plans, especially for patients in rural or underserved areas. By integrating home care strategies with primary care and hospital-based support, healthcare providers can reduce unnecessary hospitalizations, enhance early detection of complications, improve maternal and fetal outcomes, and optimize the quality of life for high-risk pregnant women. 

## Conclusions

This case highlights the critical importance of early recognition and prompt treatment of Wernicke’s encephalopathy in pregnancy, particularly among patients with severe HG who are at risk of thiamine deficiency. Delay in diagnosis and treatment of WE can lead to irreversible neurological damage, maternal morbidity, and poor fetal outcomes. 

This case emphasizes the need for early thiamine supplementation in pregnant women with prolonged vomiting and significant weight loss prior to the development of neurological symptoms. Furthermore, early and coordinated follow-up by the primary care practice following discharge from the hospital is essential. A multidisciplinary care approach involving obstetricians, neurologists, dietitians, and primary care providers ensures a comprehensive and holistic management, including nutritional support, electrolyte correction, and long-term monitoring. Increasing awareness of WE among healthcare professionals is vital to facilitate early detection and timely intervention, ultimately improving maternal and fetal outcomes while reducing the risk of chronic complications. This case serves as an important reminder to consider WE in pregnant patients, especially in those with nutritional deficiencies following HG, who can present with classic neurological features of this serious but preventable condition.
